# A Fire Revealing Coastal Norway’s Wildland–Urban Interface Challenges and Possible Low-Cost Sustainable Solutions

**DOI:** 10.3390/ijerph19053038

**Published:** 2022-03-04

**Authors:** Torgrim Log, Anna Marie Gjedrem

**Affiliations:** 1 Fire Disasters Research Group, Department of Safety, Chemistry and Biomedical Laboratory Sciences, Western Norway University of Applied Sciences, 5528 Haugesund, Norway; anna.marie.gjedrem@hvl.no; 2 CERIDES—Excellence in Innovation and Technology, European University Cyprus, 6 Diogenes Street, Engomi, Nicosia 1516, Cyprus

**Keywords:** Sotra fire, fire development, *Calluna vulgaris*, *Juniperus communis*, Sitka spruce, WUI fire prevention, land management

## Abstract

The *Calluna vulgaris* L. that dominated coastal heathlands of Western Europe were for millennia managed by regular burning cycles for improved grazing. Most places in Norway this practice has, however, been neglected over the last 5–7 decades, resulting in accumulation of above ground biomass including degenerated *Calluna* and successional fire-prone species, e.g., native juniper (*Juniperus communis*) and exotic blacklisted Sitka spruce (*Picea sitchensis*). Today, in dry periods, the heathland represents a fire threat to the increasing number of homes in the wildland–urban interface (WUI), as exemplified by the June 2021 Sotra Island WUI fire. The fire burned 700 ha of encroached heathlands, destroyed three buildings, and threatened settlements. In the present study, the Sotra fire was investigated to understand the fire development and analyse possible risk reducing measures. Photographic material obtained during the fire, weather conditions prior to and during the fire, involved fire fuel, fire spread mechanisms, firefighting response, and possible consequences under slightly changed circumstances were analysed. Compared to previous fires in coastal Norway, the Sotra fire represents a step change in fire development including, e.g., pyrocumulus-like clouds, fire whirls, and fire spread 270 m across a fjord. Preventive measures based on the local context are analysed, including engaging voluntary communities to remove fire-prone fuel, e.g., juniper and Sitka, to create defensible space. Moreover, strategic fire breaks in the terrain, e.g., well-managed heathland strengthening existing fuel breaks, e.g., lakes, cultivated fields, naked rock, and roads, are recommended. Mechanical cutting is suggested as a short-term measure while fenceless grazing may represent a long-term solution to prevent regrowth. During a period of record high energy prices, this may provide free of charge firewood and make way for future local food production, in line with the UN Sustainable Development Goals, while reducing the fire risk.

## 1. Introduction

During the 21st century, wildland fires represent an increasing threat in most fire-prone areas [1]. In the wildland–urban interface (WUI), they pose a hazard to people, homes, and other constructions [2,3,4]. Megafires, often defined as burning more than 10,000 ha (100 km^2^), consume large amounts of biomass, and release huge amounts of greenhouse gases to the atmosphere [5]. Due to climate change, the megafires are intensified and the fire seasons prolonged, creating increasing challenges for fire management [6,7]. “Climate-fuelled megafires” have, therefore, gained global attention. The EU identifies land use and land cover changes as the main drivers of the increasing trend in WUI fires [8]. Combined, climate and land cover changes may result in large fires striking multiple communities threatening life and structures and blocking escape routes [9,10].

During the last century, WUI fires were mainly addressed through the lens of “fire suppression/extinction” [1]. Subscribers of this discourse perceived wildfires as detrimental to the natural environment, economy, and society. Consequently, prescribed burning was generally prohibited for decades in places such as USA, Portugal, Spain, and Greece [11,12,13]. This resulted in biomass accumulation and finally, even more severe fires. Increased firefighting efforts could not maintain pace with the growing challenges. Early in the 20th century, opposing the fire exclusion discourse, light burning management tools have been promoted by a few practitioners, such as park manager J. R. White, who argued that fires had always been a part of the natural environment [13]. Excluding fire from the ecosystems would delay and intensify wildfires and increase future vulnerability. Currently, wildfire prevention stakeholders [8,14,15,16,17] highlight that traditional landscape and resource management, including prescribed burning, actually prevent severe wildfires.

Anthropocene landscape management by fire predates written history [18,19,20,21]. Along the European Atlantic coast, *Calluna Vulgaris*-dominated heathlands were managed by regular burning cycles and livestock herding for the past 5–6000 years [13,14,18]. This landscape is also important for its intrinsic value because it supports a variety of plants, insect, amphibians, reptiles, mammals, and bird species. The symbiotic human–herbivore–*Calluna* relationship led to the development of a unique species adaptation found in coastal Western Europe, i.e., smoke-adapted germination [22,23,24], not found in unmanaged *Calluna* at high-altitudes. The traditional landscape and resource management, including prescribed burning, prevented severe wildfires in these landscapes [14,15,16,17,25].

During the past 70 years, the traditional management of *Calluna*-dominated heathlands gradually ceased in coastal Norway due to changing agricultural practices [26] and better career opportunities following the 1969 North Sea oil discovery. After WW2, Sitka spruce (*Picea sitchensis* (Bong.) Carr.) forestry was introduced in coastal heathlands to support local economic growth [27]. Today Sitka is recognised as a blacklisted invasive species threatening local biodiversity. By 1997, only 10 per cent of the *Calluna*-dominated heathlands in Norway were preserved [27]. Today, the Norwegian government facilitates initiatives to resume the heathlands, backed by scientific research [28,29].

Local land abandonment led to invasion of the rapidly and uncontrollably spreading (outside plantation areas) Sitka spruce and native juniper (*Juniperus communis* L.), both highly combustible fire fuel when dry. Wildfire spreads rapidly through degenerated and dry *Calluna* heaths, which may act as ladder fuel for juniper, and both may act as ladder fuel for tall Sitka spruces. Accumulation of biomass in coastal heathlands and increased proximity to settlement in this area have gradually increased the WUI fire risk [30].

Following the oil discovery, numerous new homes were established in the WUI and the wildland–urban intermix at the Norwegian west coast. In January 2014, during sub-zero temperatures, communities in Flatanger and Frøya, who are 210 km and 320 km south of the Polar circle, respectively, experienced the consequences of neglected heathlands [31]. In Flatanger, a 15 km^2^ wildfire destroyed more structures than any single fire in Norway has since 1923 [32]. Fortunately, the soil contained sufficient humidity (ice) to prevent soil combustion and loss of the seed layer, and the heathland is currently re-establishing itself. Investigations of these fires [31,32] concluded that detrimental consequences to the seed bank, soil loss, and soil erosion, etc., could have happened had a similar fire taken place during a dry period in the summer, i.e., when the soil was more likely ignited. Log et al. [32] claim that re-establishing prescribed burning at regular intervals could mitigate the risk of WUI fires developing into destructive peat fires. In some areas along the Norwegian coast, initiatives for resuming the heathlands have recently been initiated by civic farmer groups [14,26]. In most areas, the encroachment continues unconstrained. Thus, there is a need to understand, and reduce, the increasing WUI fire risk [4]. 

The west coast of Norway has challenging weather conditions with high wind strengths and much precipitation, mostly in the form of rain. During the last few decades, new homes were generally built in the WUI and on islands, as the seaside provides opportunities for recreational activities. The detached, semi-detached, or terraced houses are generally heated by electricity, but are also commonly equipped with wood burners, a safety measure in the event of major power outages. Rural households consume more wood than urban households do [33]. Many rural homes are self-supplied with firewood from cottages or family farms to the extent that wood burning during wintertime is a part of the culture, exemplified by the expression “hel ved” (solid wood, i.e., “rock solid”) used about people you trust. Moreover, the book “Hel ved”, explaining cutting, drying, tinder, and burning, became the 2015 best-selling book in Norway [34]. Though firewood consumption has not recently been investigated as it has been in, e.g., Australia [35], the consumption in Norway is considerable due to the cold climate [36]. Much research has been devoted to regulative standards and manufacturing of wood burners regarding combustion efficiency, improved heat transfer, and reduction of exhaust gas soot particles and other pollutants [37].

Oil and gas extraction is very important for the Norwegian economy, providing 200,000 jobs with expected government income of NOK 154 billion in 2021 [38]. Since 1996, the government has invested the net cash flow from petroleum activities in the Government Pension Fund Global, today estimated at three times Norway’s GDP, to provide wealth benefits for current and future generations [39]. This industry accounts for 28 per cent of total Norwegian greenhouse emissions [40]. The policy framework on climate change aims at reducing such emissions by the “polluter pays” principle [40]. Measures are therefore taken to reduce emissions from this industry, mainly by electrification of process and transport equipment, e.g., boilers and compressors, etc. [41]. In combination with a new power export cable to the UK, this has resulted in increasing local electricity prices, with all-time-high prices in 2021. This is indeed discussed at government level in Norway [42]. The soaring energy prices may be explained by lagging development of renewable energy, low electricity production in German and Danish wind parks due to modest wind strength in 2021 and limited local hydropower production due to dry weather conditions. Thus, heating by firewood is even more attractive.

A peculiar part of the Scandinavian culture, particularly in Norway, is the concept called “dugnad”, i.e., voluntary work performed as collective or voluntary community work traditionally involving social work gatherings for a common benefit. Examples can be to support local neighbourhoods, schools, kindergartens, and sports clubs, etc., through construction work, fund raising, or renovation. Even the outbreak of the COVID-19 pandemic was successfully managed when the Prime Minister appealed to people’s “dugnad spirit”, i.e., a collective effort to beat the outbreak [43]. Dugnads are carried out independent of age, economic class, and abilities. The diversity of individuals brings together a variety of local knowledge and skills to the benefit of the community [33]. Dugnad has a long history in Norway. Dugnad can be viewed in relevance to the overall Norwegian welfare system, and still maintains significant social benefits [43]. Simon and Mobekk [33] use the concept of dugnad to highlight why the petroleum assets have provided wealth benefits for the overall society in Norway:

*“The hallmarks of the Nordic cultures are their consistent and strong advocacy of the ideal of a cooperative, tolerant, and inclusive community, which is regarded as superior to a competitive, hierarchic culture […]. Norway is one of the richest and most egalitarian democracies in the world. No doubt, the oil resources have played a major role in accumulating wealth, but […] also high degrees of equality and reciprocity significantly contribute to the success of the Nordic nations.”*

Thus, dugnad engagements may help solve local fire hazards. 

The purpose of the present study is to report on and analyse a fire that took place at Sotra island, 60.4° N, a half hour drive west of Bergen, Norway, June 2021, and to analyse possibilities for wildland fire fuel management. Emphasis will be given to photographic evidence from the fire, recorded weather data prior to and during the fire, fire fuel characteristics, and topography. Attention will also be paid to the anatomy of the fire, which introduced characteristics not experienced in previously *Calluna*-dominated heathlands. Section 2 presents the affected area and the fire illustrated by maps and field observations. Section 3 presents the methods of investigation. Section 4 presents the findings while Section 5 presents possible solutions to prevent similar and possibly more severe fires, with emphasis on fire fuel management in the defensible space and at strategic locations. The methodology, results, and suggestions for future mitigation measures are discussed in Section 6. Major takeaways are presented in the Conclusions. The Sotra fire June 2021 had many similarities with fires in areas considered far more fire prone and may be a sign of a shifting fire regime in coastal Norway. The presented results and fire mitigation measures may also have bearings elsewhere.

## 2. Øygarden Municipality, Sotra Island, and the WUI Fire, June 2021

### 2.1. Øygarden Municipality and the Sotra Island

**Figure 1:**
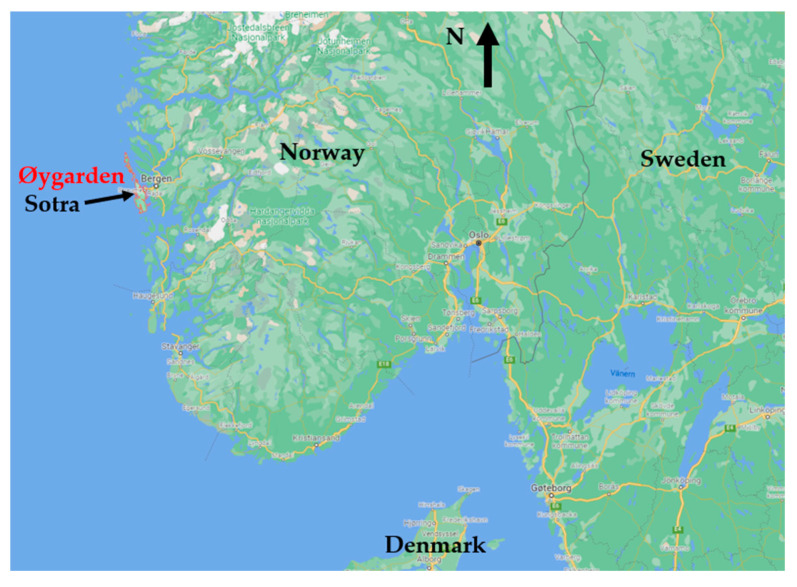
Location of Øygarden municipality and Sotra island on the Atlantic coast of Norway.

Sotra is a 176 km^2^ island at the Atlantic coast of Norway, 15 km W of Bergen city. It is 32 km long and aligned NS along the coast, see Figure 1. It mostly has a low elevation, with hills on the E side reaching 341 m above sea level (ASL). It has 19,000 inhabitants and is a major part of the Øygarden municipality with a population of 39,000 [44]. 

**Figure A1:**
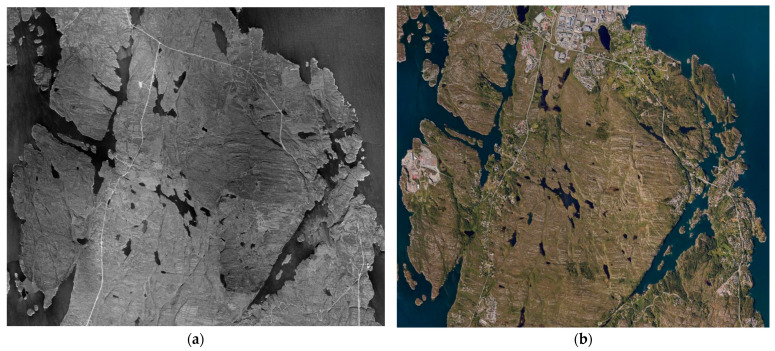
Aerial photos of the involved fire area; (**a**) 1962; (**b**) 2018. (Source: www.norgeibilder.no, accessed on 24 January 2022).

**Figure A2:**
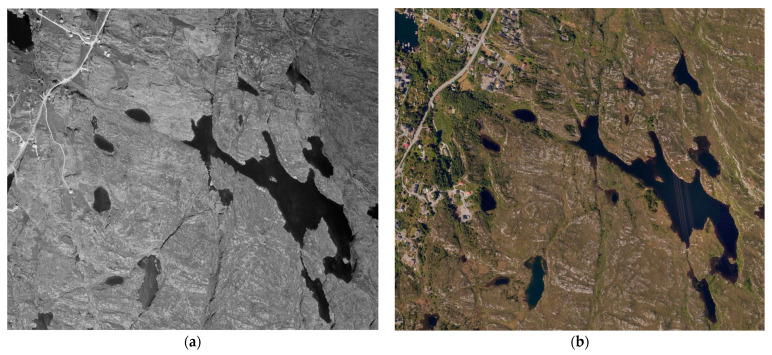
Close-up aerial photos of the central part of Figure A1; (**a**) 1962; (**b**) 2018. (Source: www.norgeibilder.no, accessed on 24 January 2022).

Until the 1950s, well managed *Calluna* heathlands dominated the island [45], see Figure A1 and Figure A2. In 1971, a 1236-m long bridge connected Sotra as a suburban area to Bergen, the second largest city in Norway. The development of the oil and gas industry from 1969, an oil terminal in 1988, and a gas processing plant in 1996 provided well-paid jobs [45]. The number of farms declined, and those left were managed by modern agricultural methods, leaving the heathlands to degrade, i.e., no livestock grazing or land management [45]. The development of new villages with schools, shopping centres, and other facilities further modernized the community. The population and numbers of settlements increased and the previous fire-safe buffer zones of cultivated farmland between homes and the wildland vanished, thus increasing the vulnerability to wildfires. 

Sotra is unique in being located within one of the major Norwegian industry regions including maritime supply bases with industrial fire trucks and trained firefighters. The main Norwegian navy base, Haakonsvern, also provides substantial resources. Moreover, proximity to, and collaboration with, Bergen fire brigade enable considerable fire response support. The accessible firefighting resources are among the top three in Norway. 

### 2.2. Weather Conditions Prior to and during the Fire

**Figure 2:**
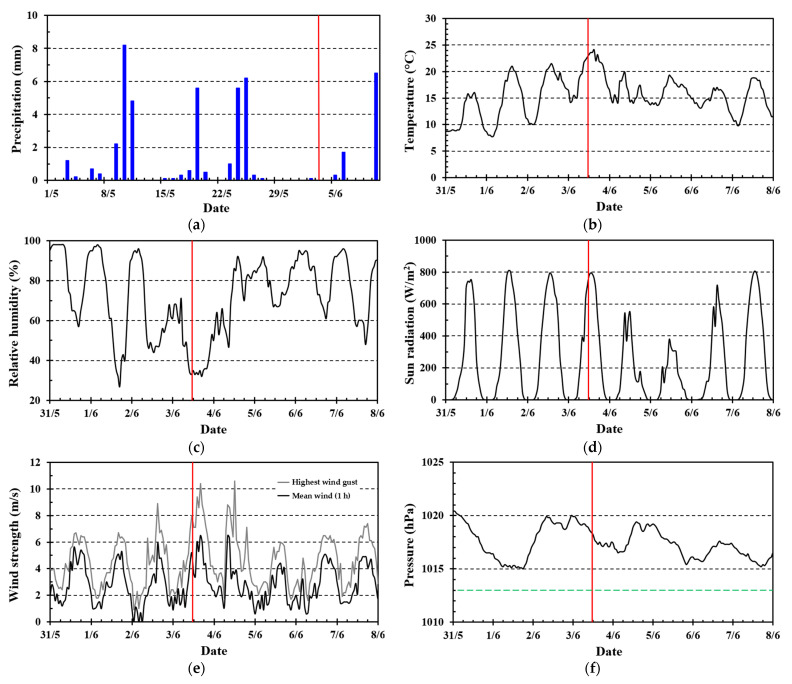
Weather parameters: (**a**) Precipitation; (**b**) Temperature; (**c**) Relative humidity; (**d**) Sun radiation; (**e**) Mean wind strength (1 h average) and strongest wind gusts; (**f**) Local atmospheric pressure (black) and neutral atmospheric pressure (green). Data from Flesland meteorological station (seklima.met.no, accessed on 23 January 2022) 13 km SSE of the fire area. (Time UTC + 1).

The weather was very dry prior to the Sotra fire, as presented in Figure 2a–f. The time of fire alarm, i.e., 11:57 a.m., 3 June (CET + 1) [45], is marked on the figures. The accumulated precipitation was only 38 mm versus expected 88 mm since 1 May. During the 8 last days, the accumulated precipitation was only 0.5 mm. 

The maximum day temperatures gradually increased to 23–24 °C, with ambient air relative humidity (RH) below 40%. The sun radiation levels peaked at 800 W/m^2^ (to a horizontal surface) resulting in local heating and corresponding low local air RH at sun-exposed biomass. Only 0.5 mm recent precipitation, low RH, and strong sun radiation for many hours at 60° N in June must have dried the vegetation considerably. It should be noted that the national fire warnings, based on the Canadian Fire Weather Index (CFWI), issued by the Norwegian Meteorological Institute, only indicated yellow fire danger.

Just after ignition, Figure 2e indicates wind speed in the range 3–4 m/s. Later, it increased to an average of about 6 m/s, gusting 10 m/s. This was possibly one of the reasons for the increase in fire spread rate and the subsequent difficulties in containing the fire. 

There were modest, high-pressure conditions, which is consistent with cloudless sky and high solar radiation levels [44]. The wind direction was from SSE (140–160°). 

### 2.3. Overview of the Sotra Fire, 3–7 June 2021

**Figure 3:**
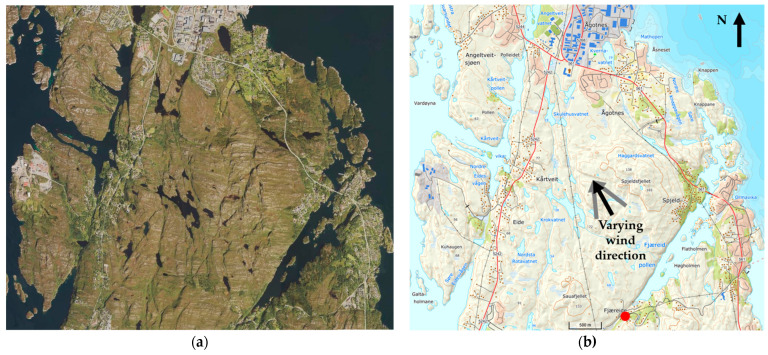
Aerial photo 4 years prior to the fire (**a**) and a map showing the ignition point at Fjæreide (marked red) and homes at risk at Eide, Kårtveit, Ågotnes, 3 June, and at Spjeld, 9 June (**b**).

An aerial photo and a map are presented in Figure 3. Scattered juniper fields, Sitka, and pine groves had established during the recent years [46], see details in Appendix A. 

**Figure 4:**
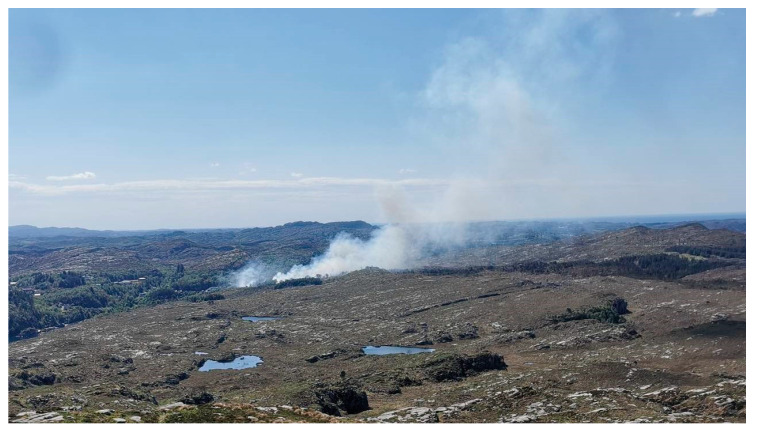
Early-stage modest fire spread. (Photo by NRK Vestland. Reproduced with permission).

The fire spread from the ignition point up a steep slope to a gravel road at 23 m higher elevation. It jumped the road and continued into the terrain. Early firefighting efforts were concentrated around protecting homes close to the ignition point and a water treatment plant to the SW. Meanwhile, the fire spread into the terrain as seen in Figure 4. To stop the fire in the terrain re-quired more resources and helicopter assistance was requested from Eastern Norway. Upon arrival at 14:00 p.m., during increasing wind strength, the experienced fire helicopter pilot immediately requested more air support. He had not experienced similar fire intensity in Norway since the 30 km^2^ Froland forest fire in 2008. 

**Figure 5:**
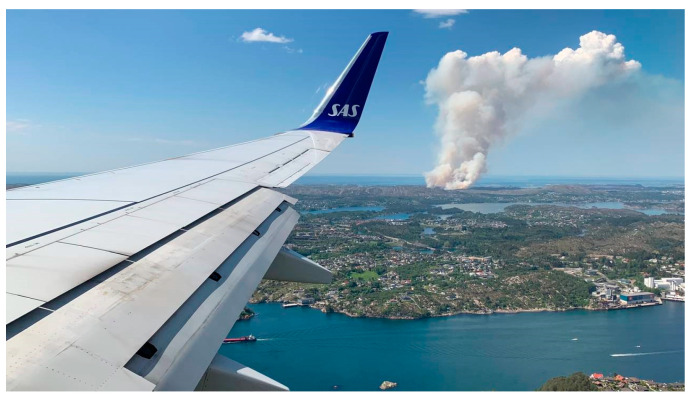
A pyrocumulus-like plume 13:30 p.m. indicated high fire intensity and modest wind conditions in the early phase of the Sotra Fire. (Photo by NRK Vestland. Reproduced with permission).

Later, the firefighters retreated due to unpredictable and intense fire behaviour, as indicated by a pyrocumulus-like plume seen in Figure 5. Left-over branches and wood from pruning below two high-voltage electrical grids, drawn as one line in Figure 2, fed the fire from south to north. The wind increased, and the fire spread towards Eide and Kårtveit. The responders adopted proactive steps to prevent the fire from reaching these communities. More than 100 firefighters and 40 civil defence personnel worked to protect homes at risk. A total of 27 fire-trucks were mobilized for firefighting and personnel transport. A firefighting (FiFi) boat and seven out of eight fire helicopters were simultaneously in operation.

Scattered homes along Vestsidevegen road through Eide and Kårtveit were threatened by the intense fire and had to be evacuated. As the fire spread northbound, it jumped Krokavatnet lake. At 17:00 p.m., the wind direction changed, and the fire spread N-NW creating a broad fire front threatening the northern communities. Ågotnes village, including a senior home, was then evacuated. Altogether, 500 people were evacuated. The fire was extinguished only 70 m from this densely populated area. Moreover, possible fire spread from ignited homes to new homes was a concern.

**Figure 6:**
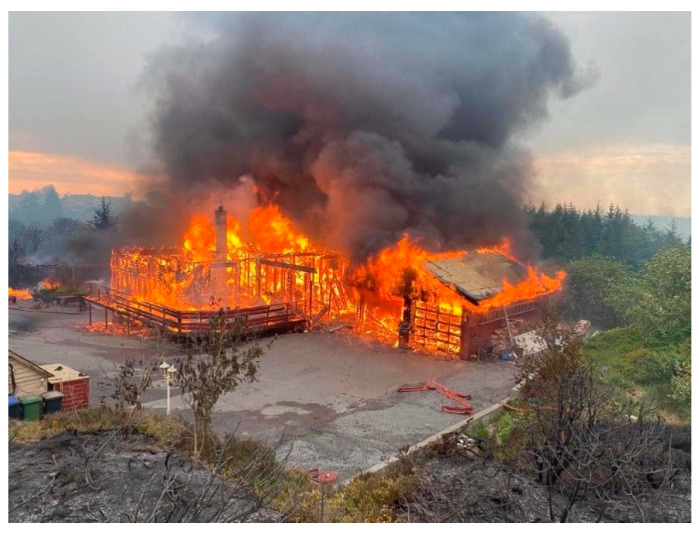
A home lost in the Sotra fire 3 June 2021. (Photo by Bergen fire brigades. Reproduced with permission).

The number of homes at risk in the WUI, the rapid-fire spread, and spotting fire made the firefighting challenging. One home, see Figure 6, and a cabin were lost while several homes suffered damage. The fire intensity and shifting winds lead to injuries and responders retreated from this home. Fire trucks and equipment were also damaged.

**Figure 7:**
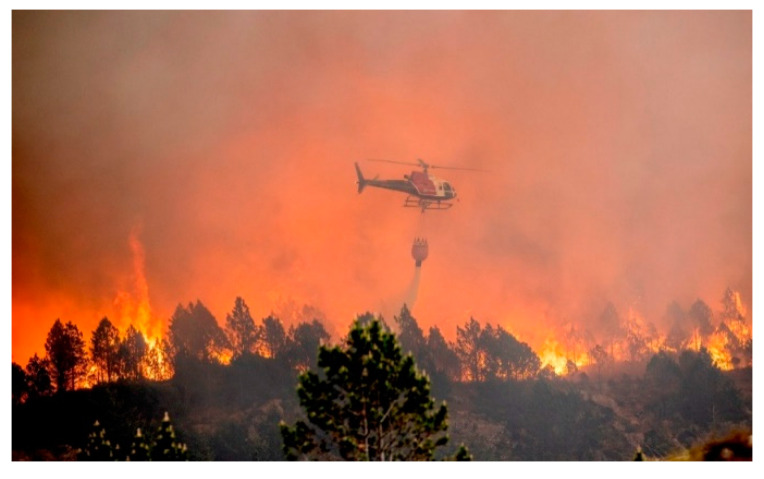
Scattered pine forest involved in the Sotra fire. (Photo by Øygarden brann og redning. Reproduced with permission).

**Figure 8:**
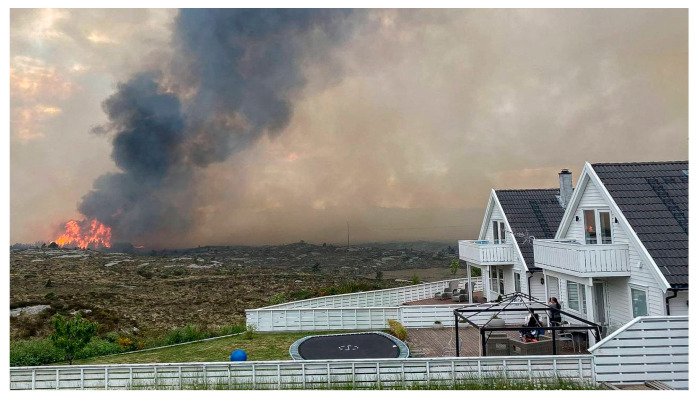
The fire approaching Søre Valderhaug, W of Skulehusvatnet lake, involving a Sitka grove. (Photo by Linnea Skare Oskarsen/NRK. Reproduced with permission).

There were two high voltage power lines, 70 kV and 300 kV, respectively, crossing the entire fire area from south to north, that were disengaged to make helicopter water drops safer, but still acted as air traffic obstructions. Frequent wind shifts hampered the fire extinguishing operations while lakes and a fjord did not work as expected fire barriers. Figure 7 shows the fire passing through a pine forest, with indications of a fire whirl. Burning Sitka groves gave flame lengths unprecedented in previously *Calluna*-dominated heathlands, creating a novel fire signature, as seen in Figure 8. 

**Figure 9:**
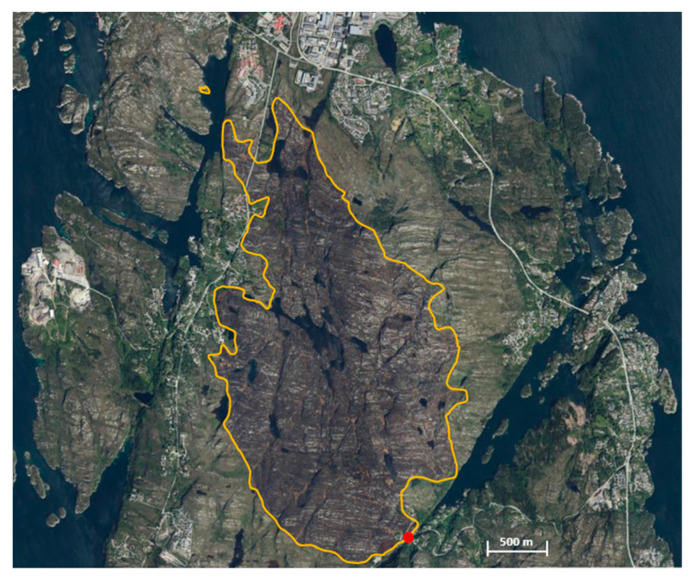
The final 700 ha Sotra fire ignition point (red dot) and footprint.

In the evening of 4 June, the evacuees were allowed to return. In the following days, visual observations and IR cameras were used to identify hot spots and smouldering nests requiring firefighting efforts. However, on the 9 June, the fire reignited in wind gusting 12 m/s. Thus, 200 inhabitants had to be evacuated as a fire front burned E towards Spjeld mountain, which was involved in a 2015 wildfire. Limited biomass resulted in less intense fire, enhanced firefighters’ mobility and safety, thus enabling extinguishing close to the mountain ridge, supported by only one helicopter. Rain during 10 June substantially reduced the risk of reignition. The fire was finally declared extinguished 13 June, due to 28 mm precipitation within this day alone. The footprint of the fire is presented in Figure 9.

No civilians were injured in the fire. However, seven firefighters were injured due to smoke inhalation and exhaustion, and spent time in medical care. Fortunately, none suffered long-term injuries. The monetary cost of the operation including equipment was about NOK 6 million. In addition, there was the cost of lost and damaged buildings, covered by insurance, and helicopter operations, covered by the Norwegian government. 

### 2.4. Why Is the Sotra Fire of Special Interest?

**Figure 10:**
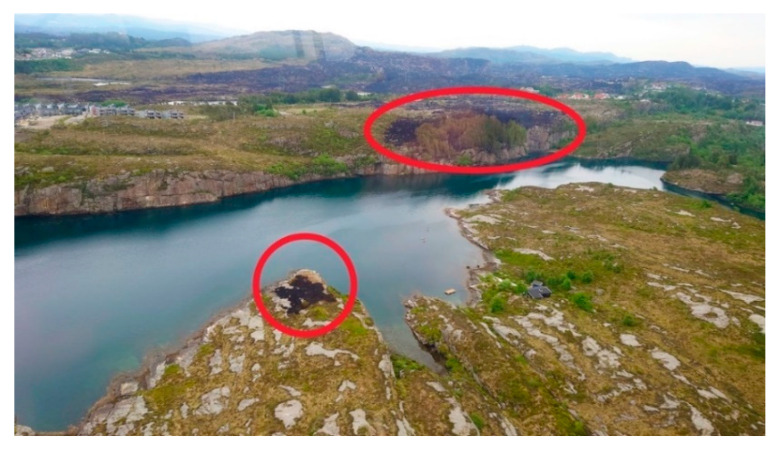
The fire jumped 270 m across the fjord Kårtveitpollen. (Photo by Øygarden brann og redning. Reproduced with permission).

Previous notable wildfires at Sotra include a 3000 ha fire in 2006, and 300 ha, 400 ha, and 500 ha fires in 2010, 2012, and 2015, respectively. The 2021 fire was, however, by far the most challenging [45]. A wildfire in previously *Calluna*-dominated heathland that resulted in fire whirls, periods of high fire spread rate and intensity, pyrocumulus-like clouds, and jumping 270 m across fjords in modest wind conditions, see Figure 10, was a new experience in a previously fire-safe landscape. The fire incident commander, on national media, willingly admitted that this fire was extremely difficult to manage, and far more difficult than the 500 ha Spjeld mountain fire 6 years earlier [47]. The fire responders also had difficulties understanding the differences between this wildfire, which was out of control with a 1 km fire front, despite fire helicopter support and massive ground crews, and fires involving only a few hectares that could easily be managed. In slightly different circumstances, e.g., stronger wind, the Sotra fire could possibly have developed into a situation similar to, or even worse than, the January 2014 Flatanger fire [32]. 

The fire helicopter pilots reported fire spread rates periodically reaching up to 4–5 m/s, i.e., unlike anything they had previously experienced. That the fire jumped 270 m over a fjord caught national attention. This jump put 250 homes, 450 m downwind, at risk. Fortunately, a firefighting (FiFi) boat had already been mobilized for reducing the fire intensity at the upwind Sitka grove, as marked in Figure 10, and could effectively suppress the new fire supported by a helicopter. It was extinguished after burning only 900 m^2^.

**Figure 11:**
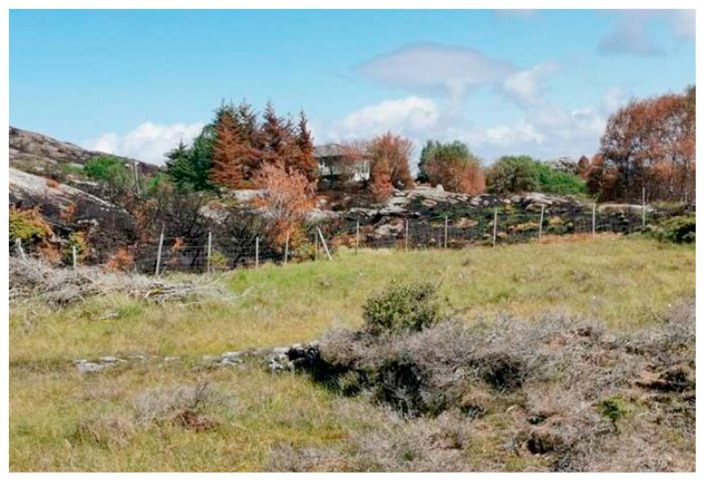
An agricultural field as a semi-natural fire break in the Sotra fire, without extinguishing efforts initiated. Photo by Øygarden brann og redning. (Reproduced with permission).

Interestingly, examples of well-functioning fire breaks were also observed during the Sotra fire. As seen in Figure 11, a field regularly grazed acted as an efficient fire break. 

The Sotra fire showed clear signs of a new fire signature in previously *Calluna*-dominated heathlands in Norway. While aged *Calluna* stands were the driver of the fires in Flatanger and Frøya, January 2014 [32], the driver for the fire at Sotra was the much more developed encroachment of fire-prone juniper and Sitka spruce. It was therefore of interest to possibly reveal insight in evolving wildfire danger indicators. A better understanding would also help in suggesting possible fire hazard mitigation measures.

## 3. Methods of Investigation

Various approaches were taken to study the Sotra fire. Personnel involved were interviewed. Photos taken during the fire by firefighters, helicopter pilots, media, and private persons were collected and analysed. Topographic and vegetational maps were studied. Field trips to the burned and adjacent unburned areas were undertaken to evaluate burn damages, fuel involved, and other signatures of the fire. Soil depts were recorded where appropriate. Meteorological data were collected from Bergen international airport, and from the fire brigades. These observations were analysed to reveal the fire signatures. Field trips were also taken to lost buildings and to buildings at risk if the fire had progressed only short distances from where it was finally extinguished.

Based on the findings, ideas for fire-prone biomass management are suggested and evaluated based on local conditions, culture, cost, and efficiency. This involves possible innovative and sustainable approaches to biomass removal and grazing, etc. 

## 4. Findings

### 4.1. The Ignition Point and the Proximity

**Figure 12:**
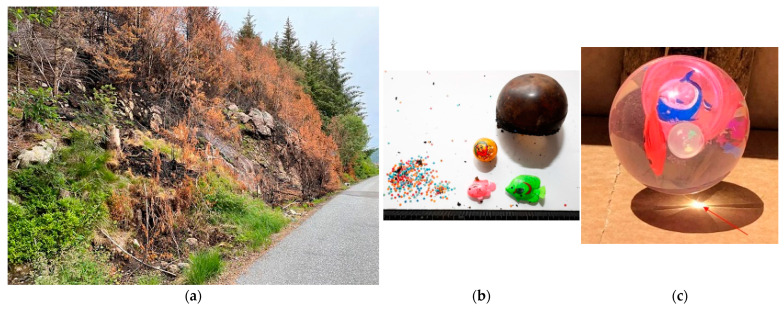
(**a**) The SE-facing steep slope where the Sotra fire started 16 m ASL; (**b**) the likely ignition source (plastic “Flash bouncing ball” with content removed); and (**c**) a similar plastic ball demonstrating the sun radiation lens effect. (Photo (**b**) and (**c**) by The National Criminal Investigation Service, Norway. Reproduced with permission).

The fire started at Fjæreide, see Figure 2, at the base of a SE-facing steep slope at 16 m ASL. The fire hearth and likely ignition source (70 mm diameter “Flash bouncing ball”), are shown in Figure 12. A similar ball acting as a magnifier lens is also shown in the figure.

**Figure 13:**
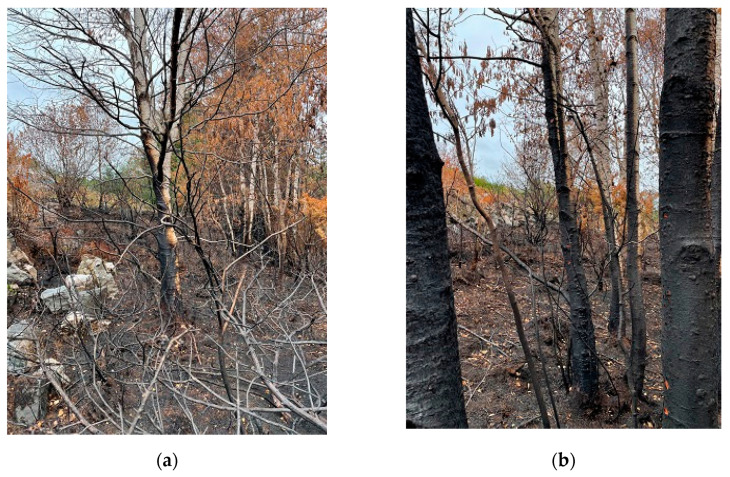
Birch trunks scorched by the fire at the plateau 20 m elevation above the ignition point: (**a**) Remains of juniper explaining combustibles able to scorch the birch trunks; (**b**) Severely scorched birch trunks.

Excavations revealed a low intensity start fire. Deciduous trees had limited burn marks with brown, but for the most part, unburned leaves. From a plateau dominated by juniper and birch at 36 m ASL, the fire had jumped a 3 m wide gravel road. Birch trunks scorched to 20–30 cm height were scorched to 4+ m on surfaces facing 2–3 m tall juniper remains, see Figure 13. These extremely combustible plants explained why the fire jumped the road. This was the first strong sign of the role played by junipers in the fire spread. 

During interviews, it was confirmed that warnings better aligned with the real fire danger would have triggered alertness and increased emergency manning. The interviewee stated that such a fire warning could provide an incentive for helicopters stationed closer in high-risk periods. It is unclear why the CFWI-based fire warnings only indicated yellow warnings, i.e., why they underpredicted the fire danger. This model is, however, developed for boreal forests while juniper and Sitka spruce on thin soil layers may require an adapted model. It could also be that models based on first principles, e.g., water vapor pressure deficit (VPD), could be appropriate [48].

The fire brigade confirmed that fire development in areas with much juniper and Sitka spruce made firefighting very challenging, and that firefighters were trapped and at significant risk when trying to protect one home. Thus, they expressed interest in initiatives regarding defensible space management. Less severe fire development in previously burned areas made them acknowledge possible prescribed burning. “An army of goats” or “dugnads” for fire fuel removal in the defensible space were also suggested.

### 4.2. Fire Spread beyond the Gravel Road

**Figure 14:**
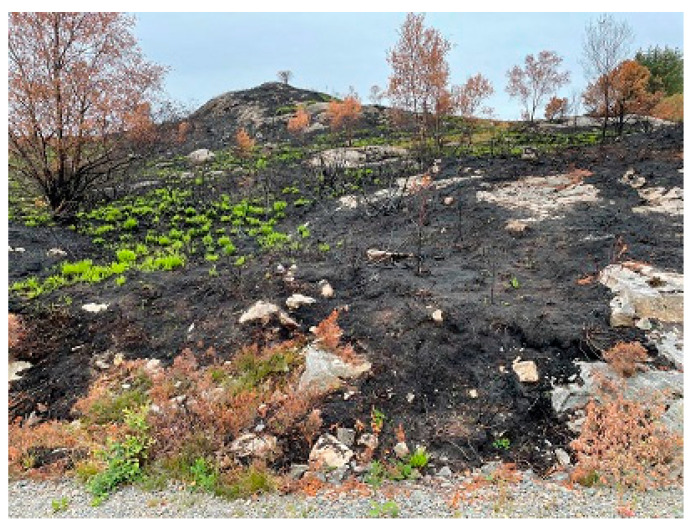
The area where the fire spread across the gravel road. This area was burned in a fire in 2015. (The W side of the gravel road is visible at the lower part of the figure).

The area just above the gravel road was exposed to a wildfire in 2015. Unburned and burned vegetation, see Figure 14, indicated a limited intensity fire caused by cured grass and young *Calluna*. 17 days after the fire, the green grass was already 5+ cm tall, indicating limited fire intensity. The organic soil layer depth varied from zero, i.e., naked rock, to 7–15 (17) cm. Such shallow soil dries quickly and makes the biomass susceptible to fire.

**Figure 15:**
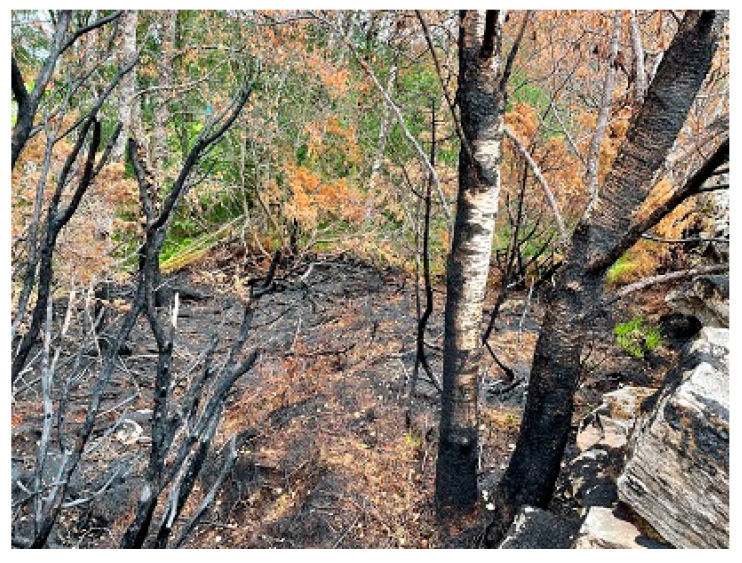
Remains of junipers to the left, partly scorched birch trunks and stone fence to the right with humid unburned vegetation in the background. (The photo is taken facing NE).

50 m to the right (NE) of the gravel road, a 1.5 m tall stone fence, where intact, represented a barrier to the fire spread. However, at locations where it was penetrated by juniper branches, biomass on the other side ignited, see Figure 15. In the more humid terrain below the stone fence, the fire spread was slow and extinguished by firefighting efforts.

**Figure 16:**
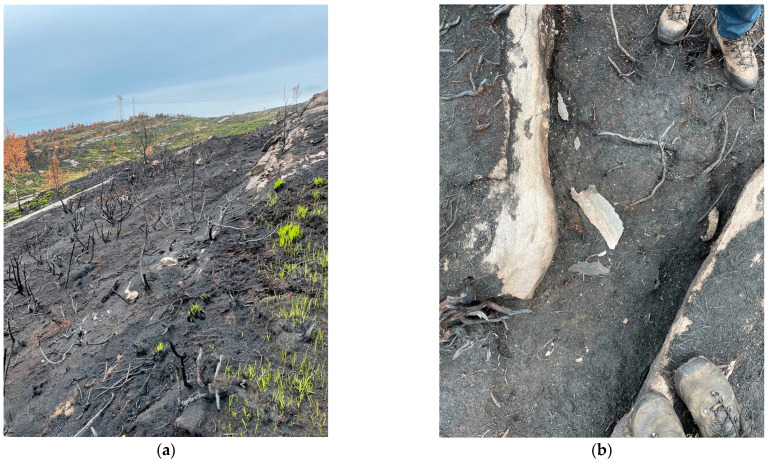
A 10 m wide and 40 m long juniper field, at 70 m ASL, 200 m due W of the gravel road: (**a**) Overview of the burned juniper field; (**b**) Rock chips (the largest about 15 cm by 5 cm) spalled by the intense juniper combustion.

Further uphill, on the S-SE-facing slope, a field of completely burned-out juniper remains were observed, see Figure 16a. Completely charred 50 mm diameter juniper remains and spalled rock chips and no regrowth indicated an intense heat exposure, see Figure 16b. This was the second sign of the role played by junipers in the fire.

For certain areas, helicopter pilots reported fire spread rates up to 4–5 m/s. This rate could possibly be anticipated in dry juniper fields aligned with wind direction in gently sloping terrain. This juniper field was one of the few places in the terrain where soil depths up to 30 cm were occasionally recorded. (It should, however, be mentioned that peat bog soil depths were not recorded as they did not play an important role in the fire spread with only partly burned vegetation). Juniper coverage continued another 50 m uphill, but only 1–2 m in width. There, less signs of ground level heat exposure were observed, likely due to less optical flame thickness and lower view factor to the ground. In several spots, ash from burned soil was observed, with seemingly randomly distributed “cavities” indicating 5–10 cm soil loss. 

### 4.3. Fire Signature in the Vicinity of one Selected Home Lost in the Fire

**Figure 17:**
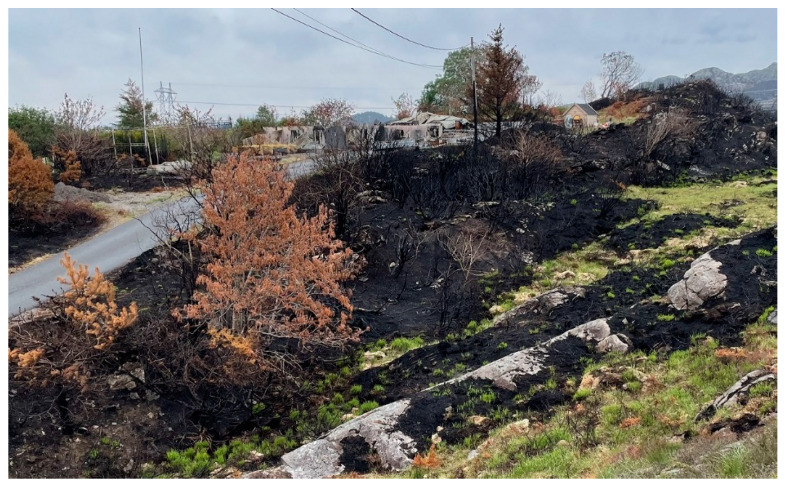
The 2–3 m tall juniper remains in a S-facing slope close to a home lost in the Sotra fire.

One home was lost in the Sotra fire, see Figure 17. The ground in the low point upwind had patches of unburned vegetation due to firefighting efforts. There were 2–3 m tall remains of junipers along the driveway and an 8 m tall Sitka spruce. The electricity grid pole was scorched by junipers up to 6 m height. Unfortunately, the firefighters had to abandon this home due to the intense heat exposure, smoke, embers, and firebrands. 

Another issue making the situation challenging is the building ventilation requirements in the generally humid climate. The prevailing western winds are adiabatically cooled when forced up by the inland mountain ranges at 1300–1600 m ASL. This results in 1300–2500 mm annual precipitation. In the fall season, humid air from the open sea condenses over land cooled by low ambient temperatures and night sky radiative cooling. Thus, the Norwegian homes have well-ventilated roof constructions, i.e., a possible access for embers and firebrands when exposed to fire. This was the main source of fire spread to homes in the studied fire as well as the previous large fires [4,32,47], and needs to be better handled in the building codes, e.g., based on experience elsewhere.

### 4.4. Impacts of Sitka Spruce on the Fire Development

Valderhaugane, at 38–42 m ASL, was forested by 20 m tall Sitka spruces, see Figure 10. Unburned Sitkas close to the sea front show that the FiFi boat had an impact. With this boat in place, and a great need for resources elsewhere, no firefighters were located at the west side of Kårtveitpollen, i.e., on the lower part of Figure 10. 

The field trip revealed several completely burned Sitka groves, while others, close to homes, had been successfully protected by firefighting efforts. With flame lengths 2–3 times the height of the tall Sitkas, e.g., as seen in Figure 8, this was indeed frightening to civilians during evacuation. Such Sitka groves resulted in spotting fire spread several places in the terrain. It came as a surprise that the fire spread 270 m across the fjord, i.e., a fire signature not previously observed in the Western Norway heathland fires.

### 4.5. Biomass Accumultion near Homes Just Outside the Burned Area

**Figure 18:**
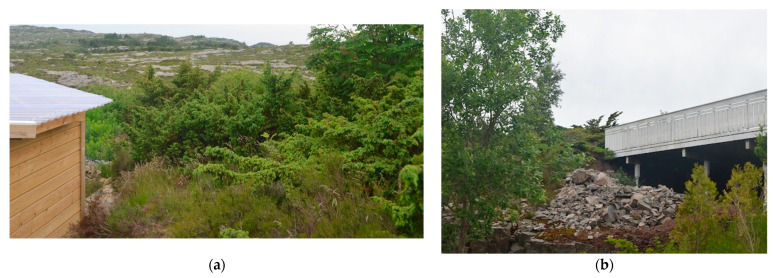
Vegetation dominated by junipers close to wooden constructions 500 m downwind from the jumped fire: (**a**) Junipers close to, and *Calluna* touching, a garage; (**b**) Junipers touching a south-facing wooden balcony.

If the 900 m^2^ burned area on the west side of Kårtveitpollen, Figure 9, had not been extinguished, 250 homes 450 m downwind would have been at risk in an area with scattered junipers close to homes facing south, i.e., upwind. Field inspections revealed virtually no defensible space as junipers touched wooden constructions, see Figure 18. Patches of 2 m tall junipers were also found within the closest 100–150 m upwind of these homes. This dense fire-prone biomass is apparently not recognized as a fire hazard.

### 4.6. Summary of the Findings

Completely charred 50 mm diameter remains of junipers, birch trees scorched by junipers, and rocks cracked by juniper combustion demonstrated that encroachment by *Juniperus communis* played an important role in the spread and intensity of the Sotra fire. 

Tall flames in Sitka groves, pyrocumulus-like clouds, and 270 m fire spread over a fjord, indicate that the fire hazard along the Norwegian west coast has changed considerably. Moreover, the FiFi boat fighting the wildland fire spreading across the fjord was an exotic and successful approach.

In several previous fires in Western Norway, *Calluna*-dominated vegetation burned without igniting Sitkas. Laypersons then assumed Sitkas were not easily ignited [49]. This may, however, be explained by previously limited ground-based fuel burning without sufficient flames and exposure time to ignite Sitka. Today, accumulated juniper and old *Calluna* represent ladder fuel. To the knowledge of the authors and the Directorate of Civil Protection [31,32], the Sotra fire is the first fire where Sitka spruce played an important role in the fire spread. Given further biomass accumulation and increasing numbers of Sitka groves, the fire hazard is rapidly increasing, as evidenced by the Sotra fire.

Fire whirls initiated by scarcely populated native pine groves also indicate that these trees were very dry and highly combustible. 

## 5. Possible Solutions to the Increasing WUI Fire Risk

### 5.1. Guiding Framework for Possible Local Sustainable Solutions

Previous research has identified degenerated *Calluna* heathlands as an increasing fire risk [4], causing fires to develop at alarming rates also in sub-zero temperatures [32]. The condition of the heathland was also a driver at the Sotra fire. Juniper and Sitka spruce encroachment severely influenced fire spread across roads and bodies of water, and to exposed buildings. Low-cost solutions for biomass reduction were therefore analysed in the present study, in line with sustainable development (SD) defined by the 1987 Brundtland Report [50] as “*development that meets the needs of the present without compromising the ability of future generations to meet their own needs*”. The SD concept emerged in response to growing concerns regarding environmental destruction and climate change. Many organizations and sectors have since adopted the concept [51]. 

The UN’s Sendai Framework for Disaster Risk Reduction 2015–2030 [52] perceives Anthropocene destruction of earth systems as a threat to society through increased natural disaster risks. Adopting SD resilience into policies, plans, programs, and budgets for disaster risk reduction is stressed. International organizations, e.g., the UN, are often governance-based, prompting intersectoral collaboration. The Organization for Economic Co-operation and Development (OECD) [53] points to successful sustainable and equitable solutions within the scope of experimental innovation and highlights that small to medium enterprises (SMEs) should be supported by big players, e.g., national governments. Enabling bottom-up initiatives is perceived as beneficial as local actors are more attuned to contextual opportunities and limitations. Grassroot SD initiatives may be more concerned with social and environmental externalities typically excluded by the current dominantly profit-based economic system. Grassroot innovators utilize available knowledge, tools, and systems to introduce incremental innovation—a concept termed “bricolage” [54]. Experimental innovation allows room for risk-taking and failure. In response to the Sotra fire, there is an opportunity for coastal communities in Norway to learn and adopt initiatives in line with the UN’s Sustainable Development Goals (SDGs) to prevent unwanted WUI fires. Thus, actions that may support the following SDGs are analysed: 11. Sustainable cities and communities; 13. Climate action; and 15. Life on land. Understanding the historical institutional context may be fruitful to highlight solutions and obstacles for such initiatives [55]. Norway’s cultural, environmental, political, and economic background possibly enabling opportunities for turning dangerous fire fuel into, e.g., valuable firewood and food, are therefore investigated. 

### 5.2. Wildland and WUI—Accessible, Productive, and Fire Safe?

Norwegians are accustomed to outdoor life. Hiking, jogging, and hunting are common activities. The open “cultural” landscape is a part of the national heritage. Thus, keeping the landscape open, or at least partly open, is highly valued [49]. The Sotra fire indicates loss of this landscape and increased wildfire risk. Establishing defensible space can reduce the WUI fire risk. Further into the terrain, strategical zones may be developed for stopping a developing wildfire. Cutting, prescribed burning, and grazing can be facilitated to extend natural fire barriers. 

A few scattered juniper fields in an otherwise well-managed heathland do not represent a significant fire hazard. It may indeed represent a diversified natural biotope. Upright junipers are generally viewed as aesthetic plants. Prostrate junipers are on the other hand viewed as a problem for, e.g., sheep, farmers, and hunters, etc., as they develop impenetrable 1 m tall stingy blankets. Some scattered prostrate juniper fields may, however, represent safe refuges for birds and prey animals. In the USA, goats have been bred and preconditioned to eat more redberry juniper [56]. Priming and breeding goats to eat more *Juniperus communis* and Sitka spruce may help prevent unlimited regrowth of these plants in defensible spaces, within strategic fire barriers, and in previously burned areas.

**Figure 19:**
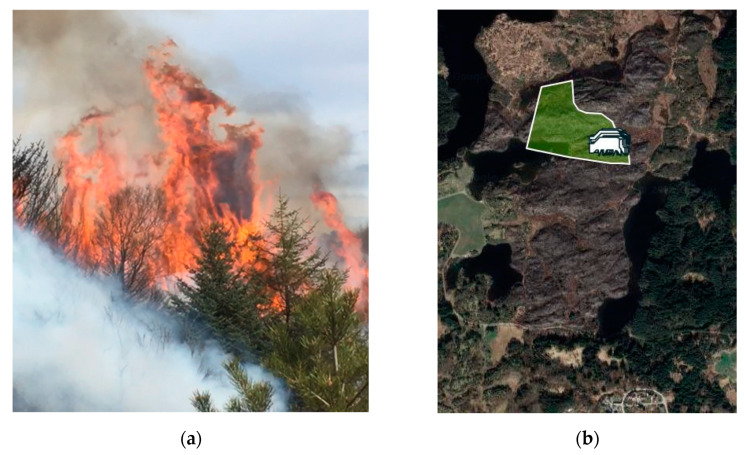
A 50 ha patch of previously *Calluna*-dominated heathland encroached by juniper, Sitka spruce, and pine at Skår, Karmøy (59.291° N, 5.256° E): (**a**) Prescribed burning 14 April 2021; (**b**) Cattle grazing wirelessly wearing GPS collars at these fields 17 September 2021.

On the 14–15 April 2021, a 50 ha prescribed burning operation was facilitated at Karmøy, Norway, on juniper-encroached heathland, see Figure 19a. Grazing cattle wearing GPS collars and guided by a virtual fencing system [57], were introduced during summer and autumn 2021, see Figure 19b. Within 2 years it is expected that pioneer *Calluna* will re-establish if exposed to a proper grazing pressure. By eliminating fencing costs, fenceless grazing landscape management is increasingly popular along the Norwegian west coast.

### 5.3. Juniper and Sitka Spruce from the WUI as Firewood?

Vegetational fire danger maps and information campaigns improve awareness. Such initiatives require time and resources [58]. Meanwhile, it is beneficial to create defensible space. Upright junipers are easily cut and chopped to firewood and recommended as tinder wood [59]. Sitka spruces, pines, and birches, etc., may also become firewood. 

**Figure 20:**
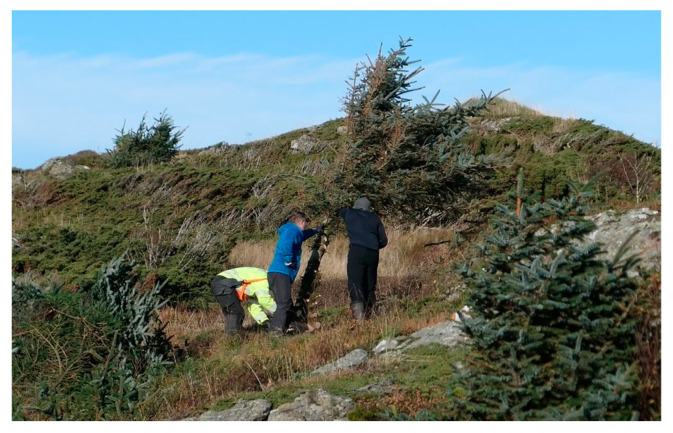
Dugnad for biomass removal at Årabrot, Haugesund, Norway, as part of a municipality heathland resumption project. Photo by Naturvernforbundet. (Reproduced with permission).

Currently, long hauled firewood and tinder wood originating from Estonia, Latvia, and Lithuania are sold at shopping centres and gasoline stations. Gradually, the value of local energy supply is appreciated by the public. There is also an increasing understanding of challenges related to vegetational encroachment paving the road for juniper and Sitka spruce removal. The municipalities of Haugesund and Karmøy, 100 km S of Sotra, currently run a project reclaiming coastal heathland between the sea front and homes [60], where locals are provided equipment for biomass removal, see Figure 20. The initiative is also supported by the County Governor and is organized as a dugnad, and the participants share the resulting firewood. A voluntary prescribed burner group studied by Metallinou [14] shall burn the area after the most fire-prone biomass is removed. Some civic groups already arrange dugnads for restoring the heathland [14,49], and these initiatives may possibly be extended to develop defensible space elsewhere, e.g., at Sotra.

### 5.4. Possibilities for Removal of Prostrate Juniper Fields

Manual removal of prostrate juniper by hand tools is challenging due to stiff tortuous stems and stingy needles. Removal may not be worthwhile if performed as a dugnad. 

**Figure 21:**
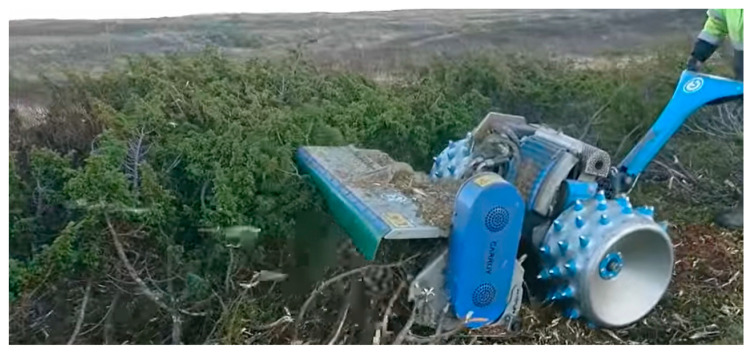
Mastication using a two-wheel tractor. Photo by Lauvrud Maskin. (Reproduced with permission).

Mastication (mulching) by excavator or tractor is costly and may harm the soil layer. Machinery adapted to two-wheel tractors may be a low-cost, low impact alternative while still being able to remove 1 m high junipers, see Figure 21. Resulting mulch can be left on site for soil conversion. The necessary machinery could be donated to non-profit organizations, or SMEs may provide professionals and machinery to perform the job. This is of special interest close to structures where prescribed burning is not a viable alternative. 

Goats eat plants not consumable by other herbivores [61] and are increasingly used for landscape management. Locally, the abundance of creeks and lakes enables unsupervised fenceless grazing. Though goats prefer deciduous trees, they also eat *Juniperus communis*. Despite that it contains monoterpenoids that may cause aversive postingestive feedback, goats may be bred for increased juniper consumption [62,63]. Preconditioning by feeding juniper to goats (and sheep) for 14 d at weaning can also improve juniper intake. Goats in groups can also be carefully conditioned to eat more juniper [56]. By feeding Boer-cross breads 0% to 30% redberry and ashe juniper, no statistical changes were observed regarding meat quality and overall acceptability among treatments [64]. 

The defensible space may be achieved quickly by cutting and mastication. Goats may be a long-term encroachment control solution. Next, the vegetation could be safely burned at regular intervals to recreate the previously fire-safe, *Calluna*-dominated heathland.

## 6. Discussion

In the present study, the fire at Sotra, Norway, 3–9 (13) June 2021, was analysed to understand drivers of the fire, and based on the findings, to suggest possible risk reducing measures. Photographic evidence and reports were collected, and conversations and interviews with personnel responsible for firefighting and evacuation, etc., were conducted. Weather conditions, prior to and during the fire, were also analysed, based on meteorological records and field observations. Field trips were performed for analysing fuel remains, soil depths, burn marks, fire spread, and vegetation next to the burned areas. 

The fire took place in dry, but not unusually dry, weather conditions. Encroachment of juniper and Sitka spruce were the most important drivers of this fuel-driven fire.

Measures to reduce the fire-prone biomass should be taken within the 30 m defensible space [65,66,67]. Householders are the agents most able to mitigate WUI fire risk through preparation and active defence [67]. Engaging householders proactively is thus very important in Norway where active defence is no option after mandatory evacuation.

High presence of efficient [37] and safe [68] wood burners in Norway and general knowledge about cutting, chopping, and drying firewood represent opportunities for utilizing upright junipers and Sitkas as firewood [34]. Heating by firewood is especially sensible during wintertime due to high electricity prices. Thus, it is recommended to initiate projects turning junipers and Sitkas into firewood. This would also limit the negative impacts of the blacklisted Sitka spreading in Norway [69,70,71]. The locals would likely work for free when keeping the firewood [60]. Afterwards, prescribed burning may resume with all its associated benefits [49,67], similar to large scale initiatives elsewhere, e.g., [72].

A recent study showed that it is cost beneficial to support land management versus firefighting [73]. Developing fire protection plans for wooden heritage buildings has been shown to give positive effects regarding fire safety [74]. Just developing municipal plans for heathland management gave reduced firefighting costs [73]. Managing areas of valuable *Calluna* heathland is already supported [75]. With much higher fire danger in neglected heathlands, funding heathland restoration would be even more beneficial cost wise. 

Regarding prostrate junipers, it would be beneficial to study the efficiency of low weight mastication equipment. An Alaskan study revealed that when neighbours mobilized for local fire fuel removal, the likelihood of other homeowners joining the initiatives increased [76]. That study may provide a framework for similar projects in Norway.

Grazing is a long-term sustainable solution for biomass control. It is highly recommended that research is undertaken to validate that goats can also be preconditioned to increase the intake of *Juniper communis* [56], which will likely represent no problem regarding meat quality [64]. Goats may make an important impact.

The study provides a dual illustration of sustainability, i.e., the signature of proposed initiatives and a systemic view [77]. The signature is low cost, low carbon footprint, and has limited negative effects on the environment. The suggested measures build on the local, historical, and institutional context including sustainability in all dimensions; environmental, societal, economic, and governance when reintroducing a fire-resilient, well-managed, semi-natural heathlands socio-ecological system (SES) that has stood the test of time. This SES may bounce back from, or reinvent itself, after shock, e.g., fire and drought [78], and support healthy and safe communities [79]. This system provided positive social and environmental (economic) externalities [80]. Re-establishing *Calluna*-dominated heathland will provide several benefits in addition to fire safety.

Well-established methods have been suggested for removing fire-prone biomass. The dugnad concept is also a well-known part of the local culture. Thus, it seems likely that the suggested measures represent solutions that can be optimized in future studies.

A fire helicopter stationed at the west coast for fast response is a very attractive suggestion. It is promising that resourceful fire brigades suggest alternative ways of solving the increasing challenges. This may pave the road for required innovation. 

The authors could not view the Sotra fire live. Conversations with involved personnel managing the fire, meteorological data, photographs, and field trips have helped to identify the most important drivers of the fire. It would be very beneficial to compare the conditions (fuel, meteorology, and topography, etc.) before and during successful prescribed burns with the conditions at the Sotra fire. This could help establishing weather based limits for safe prescribed burning of degenerated *Calluna* heathland, extending the studies performed thus far [30].

Regarding goats, the analyses rely on literature data and conversations with goat farmers. They claim that the only plant left uneaten by goats is degenerated *Calluna*, which can be burned to stimulate growth of young plants valuable for sheep grazing [49]. 

While discussed at annual prescribed fire meetings [14], strategically grazed fire barriers within the terrain is, to our knowledge, the first mention in the research literature regarding Norwegian conditions. Such barriers may be arranged as an extension to natural or manmade barriers, e.g., lakes (safe blue), naked rock (safe grey), cultivated fields (safe green), and well-managed *Calluna*-dominated heathlands (safe pink) and roads. 

In the present study, the firefighting as such was not analysed. The study was restricted to evaluate the wildland fuel, soil depths, and weather data, and to explain why the Sotra fire was difficult to manage. In addition, the study then suggested possible sustainable solutions for mitigating similar, or likely worse, wildfires in the future.

Mobilizing locals to create defensible space by turning fire-prone juniper and Sitka spruce into firewood is appealing. Fenceless grazing may then keep the biomass under control. Further into the terrain, cutting, prescribed burning, and grazing may be reintroduced at strategic locations for managing potential wildfires while still small. These initiatives support SES resiliency when faced with likely increasing future fire challenges. 

The study may be a valuable contribution towards a fire-safe future in coastal areas of Western Norway. Because similar WUI fire challenges exist in many countries [81], the suggested solutions may have validity in other regions and climate zones, e.g., where winter dry wooden homes may make the situation even worse [82,83]. 

## Figures and Tables

**Figure A3:**
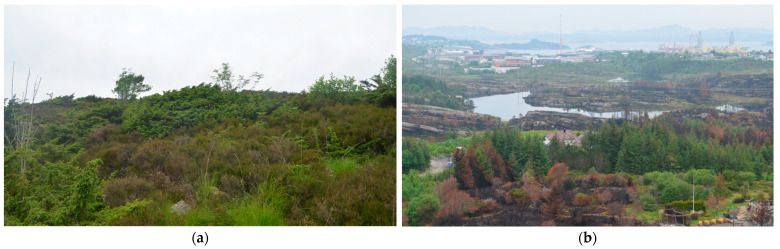
Representative fuel at Sotra; (**a**) unburned juniper and *Calluna*; (**b**) partly burned Sitka spruce forest.

## Data Availability

The presented weather data were collected from the Norwegian Meteorological Institute through the publicly available weather service: seklima.met.no, accessed on 5 January 2022.

## Appendix A

Figure A1 and Figure A2 show aerial photos of the involved area from 1962 and 2018, with significantly more vegetation in 2018. Representative photos of previous *Calluna*-dominated heathland at Sotra encroached by *Juniperus communis* and Sitka spruce are shown in Figure A3. Settlements and industry are seen in the background of Figure A3b.
